# Utilising animal models to evaluate oseltamivir efficacy against influenza A and B viruses with reduced *in vitro* susceptibility

**DOI:** 10.1371/journal.ppat.1008592

**Published:** 2020-06-18

**Authors:** Rubaiyea Farrukee, Celeste Ming-Kay Tai, Ding Yuan Oh, Danielle E. Anderson, Vithiagaran Gunalan, Martin Hibberd, Gary Yuk-Fai Lau, Ian G. Barr, Veronika von Messling, Sebastian Maurer-Stroh, Aeron C. Hurt

**Affiliations:** 1 WHO Collaborating Centre for Reference and Research on Influenza, VIDRL, at the Peter Doherty Institute for Infection and Immunity, Melbourne, Victoria, Australia; 2 Department of Microbiology and Immunology, The University of Melbourne, at the Peter Doherty Institute for Infection and Immunity, Melbourne, Victoria, Australia; 3 School of Health and Life Sciences, Federation University, Churchill, Victoria, Australia; 4 Programme in Emerging Infectious Diseases, Duke-NUS Medical School, Singapore; 5 Bioinformatics Institute, Agency for Science, Technology and Research, Singapore, Singapore; 6 Genome Institute of Singapore, Agency for Science, Technology and Research, Singapore, Singapore; 7 Veterinary Medicine Division, Paul-Ehrlich-Institute, Federal Institute for Vaccines and Biomedicines, Langen, Germany; 8 National Public Health Laboratories, National Centre for Infectious Diseases, Ministry of Health, Singapore; 9 Department of Biological Sciences, National University Singapore, Singapore; University of Wisconsin-Madison, UNITED STATES

## Abstract

The neuraminidase (NA) inhibitor (NAI) oseltamivir (OST) is the most widely used influenza antiviral drug. Several NA amino acid substitutions are reported to reduce viral susceptibility to OST in *in vitro* assays. However, whether there is a correlation between the level of reduction in susceptibility *in vitro* and the efficacy of OST against these viruses *in vivo* is not well understood. In this study, a ferret model was utilised to evaluate OST efficacy against circulating influenza A and B viruses with a range of *in vitro* generated 50% inhibitory concentrations (IC_50_) values for OST. OST efficacy against an A(H1N1)pdm09 and an A(H1N1)pdm09 virus with the H275Y substitution in neuraminidase was also tested in the macaque model. The results from this study showed that OST had a significant impact on virological parameters compared to placebo treatment of ferrets infected with wild-type influenza A viruses with normal IC_50_ values (~1 nM). However, this efficacy was lower against wild-type influenza B and other viruses with higher IC_50_ values. Differing pathogenicity of the viruses made evaluation of clinical parameters difficult, although some effect of OST in reducing clinical signs was observed with influenza A(H1N1) and A(H1N1)pdm09 (H275Y) viruses. Viral titres in macaques were too low to draw conclusive results. Analysis of the ferret data revealed a correlation between IC_50_ and OST efficacy in reducing viral shedding but highlighted that the current WHO guidelines/criteria for defining normal, reduced or highly reduced inhibition in influenza B viruses based on *in vitro* data are not well aligned with the low *in vivo* OST efficacy observed for both wild-type influenza B viruses and those with reduced OST susceptibility.

## Author summary

Oseltamivir (Tamiflu) is an antiviral widely used for the treatment of infection due to influenza viruses and is especially useful for the treatment of severely ill high-risk patients. Antiviral resistance to oseltamivir is of concern as it can undermine the utility of the drug. Influenza viruses can become less susceptible to oseltamivir due to amino acid substitutions that arise in the viral surface protein, neuraminidase (NA). During influenza surveillance, to monitor for resistance, laboratory experiments are carried out to measure viral susceptibility to oseltamivir, whereby the quantity of drug needed to inhibit NA enzyme action is measured. However, how well these laboratory measurements predict resistance of viruses to oseltamivir in a clinical setting is not well understood. In this study, we developed an animal model to evaluate this relationship between laboratory data and clinical efficacy in more detail and demonstrated that *in vivo* efficacy of oseltamivir is lower against viruses that show reduced inhibition by oseltamivir in laboratory testing. A key finding from our analysis was that oseltamivir had reduced efficacy in reducing viral shedding against wild-type influenza B viruses compared to wild-type influenza A viruses, which has implications on how data from influenza B viruses with reduced susceptibility are currently being interpreted.

## Introduction

Infection with influenza viruses results in a highly contagious respiratory disease that causes substantial global morbidity and mortality [1]. Antivirals play a major role in the treatment and prophylaxis against influenza infections and neuraminidase inhibitors (NAIs) remains the most commonly used antiviral [2, 3]. NAIs are sialic acid analogues that target the viral neuraminidase (NA) protein and competitively inhibit enzyme activity by blocking the NA active site [4, 5]. While there are four NAIs currently available (zanamivir, oseltamivir (OST), laninamivir and peramivir [6–8]), OST is the most commonly prescribed antiviral globally, due to its ease of oral administration [9].

Resistance or reduced susceptibility to OST can arise due to amino acid substitutions in the NA active site or the region surrounding the active site, that can lead to reduced drug binding [10–17]. However, the overall proportion of circulating influenza viruses with reduced OST susceptibility has generally remained relatively low (below 2%), since the drug’s licensure in 1999 [10–17]. The only exception was during the 2007–2009, when a former seasonal influenza A(H1N1) virus with the H275Y NA amino acid substitution circulated world-wide and led to guideline changes against the use of OST [18, 19]. The emergence of this virus underscored the importance of maintaining regular surveillance of influenza viruses to monitor antiviral susceptibility [10–13, 20]. However, fortuitously, this virus was replaced during the 2009 pandemic with an OST sensitive swine-origin A(H1N1)pdm09 virus [21, 22], lacking the H275Y substitution. While community outbreaks have also occurred since 2009 with H275Y A(H1N1)pdm09 viruses [23, 24], the overall prevalence of H275Y in this subtype has generally remained low.

Susceptibility of viruses to OST is typically determined using an *in vitro* NA enzyme inhibition assay to measure the 50% inhibitory concentration (IC_50_) of the drug, which is then used to infer if a virus has reduced susceptibility or not [25, 26]. While this assay is a quick, high throughput and convenient way to analyse viral isolates, early studies differed in their classification of viruses based on IC_50_ values [14, 25, 27]. To improve reporting of viruses with reduced susceptibility, a criteria was standardised in 2012 by the WHO working group on influenza antiviral susceptibility (WHO-AVWG) [28], such that influenza A viruses with a 10–100 fold increase in OST IC_50_ compared to a susceptible human influenza virus of the same NA subtype were described to have ‘reduced inhibition’, while those with greater than a 100-fold increase in OST IC_50_ were described as having ‘highly reduced inhibition’. The equivalent fold-differences for influenza B viruses were set to 5–50 fold for ‘reduced inhibition’ and greater than 50-fold for ‘highly reduced inhibition’, due to wild-type influenza B viruses having higher baseline OST IC_50_ values than influenza A viruses [28]. However, this classification system was relatively arbitrary with thresholds set to help coordinate reporting of *in vitro* enzymatic assay data between different laboratories, rather than having any clinical relevance. As such there has been no agreement or studies on how these *in vitro* IC_50_ values relate to OST treatments *in vivo*, against human influenza viruses with reduced susceptibility.

There are only a few examples where the clinical effectiveness of OST against viruses with increased OST IC_50_ values have been evaluated. In a Japanese multicentre study during the 2008–2009 influenza season, OST was administered to patients prior to knowing that the infecting virus contained the H275Y NA substitution with a highly increased OST IC_50_ [29]. Analysis of the clinical data showed that OST-treated patients infected with viruses bearing the H275Y substitution had increased fever duration compared to OST-treated patients infected with wild-type A(H1N1) viruses [29].Two other studies in Japan, also demonstrated reduced benefit of OST treatment in children infected with the influenza A(H1N1) (H275Y) virus, compared to those infected with the wild-type A(H1N1) viruses [30, 31]. There are also limited examples from two cases of immunocompromised patients infected with influenza A(H3N2) viruses bearing the E119V NA substitution with an increased IC_50_, whereby a rebound in viral load was observed following the emergence of this virus, despite continued OST treatment [32, 33]. Finally, a number of clinical studies have shown that OST is less effective in reducing symptoms in patients infected with wild-type influenza B viruses, which have higher average OST IC_50_ values, compared to patients infected with wild-type influenza A viruses [14, 34–39].

Although these ad-hoc case studies demonstrate that the efficacy of OST is reduced or lost against some viruses with increased OST IC_50_ values, the magnitude of the change is not clear and many clinical studies are confounded by underlying health aspects of the patients, such as being immunocompromised, which are likely to reduce antiviral efficacy. Therefore, our aim in this study was to assess the efficacy of OST against a range of wild-type and reduced susceptibility seasonal influenza viruses with varying IC_50_ values in different animal models and establish a relationship between the *in vitro* IC_50_ data and the *in vivo* OST efficacy.

We focussed initially on the ferret model because unlike many other animal models, ferrets can be infected directly with human influenza viruses and exhibit clinical signs similar to those displayed by humans [40–44]. Due to the lack of a reduction in the virus load seen in previous studies with OST in ferrets ‘artificially’ infected with a large intranasal dose of virus [45–47], we utilised a model of ‘natural’ infection whereby ferrets were infected by exposure to an experimentally infected donor, similar to the way in which humans are naturally infected by influenza. We further carried out a pilot experiment, testing OST efficacy in macaques infected with either a wild-type or reduced susceptibility virus. While viral replication in macaques was poor and did not produce conclusive results, we were able to evaluate the effect of OST dosing on viral shedding and clinical signs in ferrets infected with either wild-type or reduced susceptibility viruses, and showed a loss of OST efficacy (based on viral shedding), against viruses with increased IC_50_ values.

## Materials and Methods

### Viruses

Wild-type viruses (lacking known amino acid substitutions in the NA drug binding region) with typically low IC_50_ values were used in this study, including; an A(H1N1) pdm09 virus (A/Perth/265/2009), a former seasonal wildtype clone of an A(H1N1) virus (A/Mississippi/3/2001), an A(H3N2) virus (A/Fukui/20/2004), and an influenza B virus from the B-Yamagata lineage (B/Minnesota/23/2015). These viruses are referred to as H1N1, H1N1pdm09, H3N2 and B respectively, hereafter in this report. The viruses with reduced susceptibility (viruses containing NA substitutions known to increase OST IC_50_ values) were an A(H1N1)pdm09 virus with the H275Y NA substitution (A/Perth/261/2009), a clone of a former seasonal A(H1N1) virus with the H275Y NA substitution (A/Mississippi/3/2001), an A(H3N2) virus with the E119V NA substitution (A/Fukui/45/2004) and two influenza B viruses from the B-Yamagata lineage, one with the H273Y NA substitution (B/Perth/265/2015), and the other with the D197N NA substitution (B/Singapore/GP702/2015). These viruses are referred to as H1N1pdm09 (H275Y), H1N1 (H275Y), H3N2 (E119V), B (H273Y) and B (D197N) respectively hereafter.

All viruses were collected as part of the World Health Organization Global Influenza Surveillance and Response System (GISRS) or kindly provided by the Centers for Disease Control and Prevention (CDC), Atlanta, United States and the National Institute of Infectious Disease (NIID), Tokyo, Japan. The viruses were cultured in Madin-Darby canine kidney (MDCK; ATCC CCL-34) cells at 35°C, 5% CO_2_ in Dulbecco’s modified Eagle Media (DMEM; SAFC-Bioscience, Lenexa, KS, USA) supplemented with 200 mM L-glutamine, 100X MEM non-essential amino acid solution, 7.5% sodium bicarbonate, 1M HEPES buffer solution, penicillin-streptomycin solution, 2 mg/ml fungizone and 4 μg/mL of TPCK-trypsin (all from Sigma-Aldrich, Castle Hill, NSW, Australia). The infectivity titre of the viruses was quantified via viral infectivity assay and a 50% tissue culture infectious dose (TCID_50_) calculated as described by Reed and Muench [48]. The haemagglutinin (HA) and NA genes of all viruses were sequenced, and their susceptibility phenotype assessed by NAI susceptibility assay as described below.

### Fluorometric NA inhibition assay

A fluorescence-based assay utilising the substrate 2’-(4-methylumbelliferyl)-a-D-N-acetylneuraminic acid (MUNANA; Sigma-Aldrich, Castle Hill, NSW, Australia) was used to measure the enzymatic activity and the relative inhibition of enzyme activity by oseltamivir carboxylate (Carbosynth, UK) using methods described previously [26]. The NAI concentration that inhibited 50% of NA activity (IC_50_) was determined using JASPR (version 1.2, CDC, USA) software program.

### Ethics statement

Experiments using ferrets were conducted with the approval of Melbourne University Animal Ethics Committee (project license number 1313040) in strict accordance with the Australian Government, National Health and Medical Research Council Australian code of practice for the care and use of animals for scientific purposes (8^th^ edition). Ferret studies were conducted at the Bio-Resources Facility located at the Peter Doherty Institute. The experiments with Cynomolgus macaques (*Macaca fascicularis*) were approved by the SingHealth Institutional Animal Care and Use Committee of the Sing Health Experimental Medicine Centre (SEMC IACUC Reference: 2014/SHS/922).

### Ferrets

Outbred approximately 6-month-old adult male and female ferrets (Animalactic Animals & Animal Products Pty Ltd, Victoria, Australia) weighing 608–1769 g were used. Serum samples from ferrets were tested by hemagglutination inhibition assay to ensure seronegativity against currently circulating influenza A subtypes and B-lineages. Ferrets were housed in high efficiency particulate air filtered cages with *ab libitum* access to food, water and enrichment equipment throughout the experimental period. Ferrets were randomly allocated to experimental groups.

### Model setup for evaluating oseltamivir efficacy

An outline for the experimental model used in this study is illustrated in S1 Fig. Ferrets were dosed orally with 5 mg/kg dose of oseltamivir phosphate (kindly provided by F. Hoffmann-La Roche AG, Basel, Switzerland) twice a day, which is considered equivalent to the standard treatment human adult dose of 75 mg/kg delivered twice daily [49]. Oseltamivir phosphate solution was prepared at a concentration of 10 mg/mL in a sterile 0.5% sugar/phosphate buffered saline (PBS) solution, while a placebo solution was prepared containing only sterile 0.5% sugar/PBS solution. On Day 1 naïve test ferrets were orally delivered either 5 mg/kg of OST or placebo and after 2 hours were exposed to an experimentally infected donor. The donors were experimentally infected on Day 0 with 500 μL of 1 x 10^6^ TCID_50_ of virus as previously described [50]. The test ferrets were dosed again with either OST or placebo 8 hours post first dose and twice daily for the next 10 days. Daily nasal washes were collected from the test ferrets and activity, weight and temperature monitoring were carried out each day at approximately the same time in the morning.

This experimental model was utilised to evaluate OST efficacy against nine different viruses, with the only variation in protocol being the exposure time of test ferrets to donors. Test ferrets exposed to H1N1-infected donors were co-housed for 48 hours, while those exposed to H3N2-infected ferrets were co-housed for a maximum of 6 days and ferrets exposed to influenza B-infected donors were co-housed for 11 days. The difference in length of co-housing was informed by previous pilot experiments that demonstrated a difference in time of transmission for each type/subtype. At the end of the experiment, ferrets were euthanized by administration of anaesthetic by intramuscular injection [50:50 mix of Ilium Xylazil (Xylazine, 20 mg/mL): Ketamine (100 mg/mL)] followed by an overdose of pentobarbitone sodium (Lethabarb, 0.5 mL/kg) (Virbac, Australia). Cardiac bleeds were taken for serological analysis by hemagglutination inhibition assay.

### Ferret monitoring, activity measurement and sample collection

All animal measurements were collected in a blinded manner. Activity levels of the ferrets were determined by video-monitoring, and body temperature and weight were measured daily at approximately the same time points as previously described [50]. Nasal washes were collected daily from sedated ferrets (intramuscular injection of Xylazine at 5 mg/kg) by instilling 1 mL of sterile PBS into one nostril and allowing the liquid to flow out of the other nostril into a collection tube. The number of viable cells in the nasal washes was counted immediately after collection. Aliquots of nasal washes were stored at -80°C with 0.01% of bovine serum albumin for determining viral titres or -20°C prior to determining protein concentrations.

### Inflammatory cell and protein concentration, virological and sequencing analysis

Viable cells in the ferret nasal washes were determined by Trypan blue exclusion using a Countess automated cell counter (Life technologies, Carlsbad, CA, USA). Protein concentration in the nasal washes were determined using Coomassie Plus (Bradford assay) according to manufacturer’s instructions (ThermoFisher scientific, Scoresby, Victoria, Australia). Titres of infectious virus in the nasal washes were quantified by a viral infectivity assay and a TCID_50_ calculated as described by Reed and Muench [48], with a minimal detection limit of 10^2^ TCID_50_/ml. Next generation sequencing was performed on nasal washes from peak shedding day from each ferret, to ensure the genetic stability of the NA gene in viruses post-transmission and following replication in ferrets, as described previously [51]. Briefly, viral RNA was extracted using QIAamp Viral RNA kit (Qiagen, Germany) and influenza genes were amplified utilising previously described primers for influenza B viruses [52] and influenza A viruses [53]. Next Generation Sequencing (NGS) was done off-site on the Illumina MiSeq platform. NGS reads were mapped to their respective genome using Bowtie2 [54] and percentage of single nucleotide polymorphism (SNPs) estimated utilising Vascan2 [55].

### Evaluating OST efficacy in macaques

Oseltamivir efficacy against the H1N1pdm09 and H1N1pdm09 (H275Y) viruses was also evaluated in a macaque model of infection. Cynomolgus macaques were purchased from the SingHealth colony and were free of antibodies against influenza as determined by immunoperoxidase monolayer assay [56]. The animals were group housed until the beginning of the experiment. During the experiment, animals were housed individually to accurately assess clinical signs and disease severity in each animal. Cages were positioned to ensure visual contact with other animals. Toys were provided for enrichment and rotated at least every week. Food was provided *ad libitum* and fruits offered as treats.

Starting the day before infection and daily throughout the study, animals were anesthetized by intramuscular injection with ketamine (10 mg/kg) and then intubated to collect throat and nasal swabs and to perform nasal and tracheal lavages. Anaesthesia was maintained with isoflurane as needed. The day before infection, a pill-size temperature sensor was given by pill applicator to record the body temperature every 15 min. If this sensor was excreted during the study, it was reapplied the following morning during anaesthesia. For the infection, three animals per group were infected via the intra-tracheal route with 4x10^6^ TCID_50_/ml. Animals were treated with 3 mg/kg OST (Tamiflu suspension, Roche) or an equal volume of fruit juice orally, starting a day prior to infection and continued for four days after. The macaques were observed daily for activity and clinical signs. Each time the animals were anaesthetized, body temperature was also measured rectally and the weight recorded. Viral titre in the different samples was quantified by the TCID_50_ assay, and next generation sequence analysis was carried out on Day 4 tracheal lavage samples, as previously described [51]. At the completion of the experiment, a necropsy was performed on each animal. Anaesthesia was followed by pentobarbital (85mg/kg) overdose through IV catheter. Death was verified and confirmed by an SEMC/NLARF veterinarian prior to necropsy.

### Statistical analysis

A non-parametric Wilcoxon rank-sum test was used to compare each individual metric: viral titre, length of viral shedding, activity, cell-count, protein concentration, weight and temperature, between ferrets/macaques in the placebo and OST group, for each day. Area under the curve (AUC) was calculated for viral titre and clinical symptoms in Graphpad prism 6.0. Difference in AUC for viral titre or symptoms between placebo and OST dosed ferrets was standardized to total AUC of the placebo dosed animals for each virus, and the spearman correlation was calculated between % differences in AUC (%ΔAUC) to IC_50_ values. The relationship between %ΔAUC difference in viral shedding and IC_50_ values were further explored by fitting different linear and non-linear models in R software [57], and the model with the lowest Akaike information criterion (AIC) value was selected. The fitted curve was plotted on R and bootstrapping was used to calculate 95% confidence intervals.

## Results

The effect of OST dosing on viral shedding in the upper respiratory tract of ferrets was evaluated by measuring a number of parameters, including a) number of infected ferrets, b) peak viral loads in the nasal wash, c) duration of viral shedding and d) area under curve of viral shedding curves (Table 1). The clinical signs evaluated during the experiments included measuring changes in body weight, temperature and activity, and cell counts and total protein concentration in nasal washes, as indicators of nasal inflammation. Changes in each clinical sign were assigned scores as shown in Table 2, which was then used to calculate a total clinical score for each test group (Table 3).

**Table 1:** The IC_50_ values of infecting viruses and viral shedding characteristics of ferrets dosed with either placebo or oseltamivir.

Influenza Virus	IC_50_^#^^$^ (fold difference)^a^	% Ferrets Infected (no.)		Peak Viral Titre^#^^$^		Duration of Shedding^$^		AUC^#^^B^	
		*Placebo*	*OST*	*Placebo*	*OST*	*Placebo*	*OST*	*Placebo*	*OST*
**Influenza A**									
**H1N1**	0.9 ± 0.4	75 (3/4)	0 (0/4)	4.4 ± 0.2	N/A	5.7±1.1	0.0 ± 0.0*	17.2 ± 12.3	0.0 ± 0.0*
**H1N1 (H275Y)**	414.8 ± 46.2 (460)	100 (4/4)	100 (4/4)	4.8 ± 0.6	4.6± 0.5	4.8±0.5	5.2±1.25	16.9 ± 0.7	18.2 ± 4.3
**H1N1pdm09**	0.3 ± 0.2	100 (4/4)	50 (2/4)	4.8 ± 0.7	4.2 ± 0.9	4.8±0.5	3.5±0.7	18.6 ± 2.9	9.3 ± 12.7
**H1N1pdm09 (H275Y)**	192.0 ± 46.9 (640)	100 (4/4)	100 (4/4)	5.5 ± 0.4	5.2 ± 0.6	6.5±1.7	6.0±1.4	24.6 ± 5.5	23.4 ± 4.4
**H3N2**	0.2 ± 0.1	100 (7/7)	50 (4/8)	4.4 ± 0.4	3.0 ± 0.8*	5.0±0.6	2.0±0.8*	14.4 ± 1.8	3.0 ± 3.7*
**H3N2 (E119V)**	65.6 ± 28.9 (328)	62 (5/8)	62 (5/8)	4.7 ± 0.9	4.8 ± 0.4	4.0±1.6	3.8±0.5	7.9 ± 7.0	6.0 ± 7.7
**Influenza B**									
**B**	36.1 ± 5.4	100 (4/4)	100 (4/4)	4.7 ± 0.9	3.7 ± 0.4	4.8±1.0	5.5±0.6	19.8 ± 4.0	12.9 ± 1.9
**B (D197N)**	66.2 ± 7.5 (2)	75 (6/8)	100 (4/4)	4.4 ± 0.8	4.5 ± 0.5	5.5±0.5	4.2±1.0	11.5 ± 6.9	12.6 ± 2.3
**B (H273Y)**	96.7 ± 10.8 (3)	100 (4/4)	100 (4/4)	4.2 ± 0.7	3.8 ± 0.6	4.0±1.1	5.2±0.5	11.3 ± 2.1	13.5 ± 1.0

^#^ Data is presented in the following format where possible: Mean ± Standard Deviation

^$^ IC_50_ unit is nM, Viral titre unit is Log_10_ TCID_50_/ml, Duration of shedding is days, and no unit for AUC

*A significant difference is observed between placebo and OST (Wilcoxon rank-sum test) dosed ferrets

^a^ Fold difference of each reduced susceptibility virus is calculated in comparison to corresponding wild-type.

^b^ Total area under the curve of raw data, therefore no adjustments were made for uninfected ferrets or varying days of first infection

**Table 2:** The clinical score card used to determine severity of clinical signs in ferrets infected with influenza viruses.

Score	% Weight loss from baseline^#^*	% Activity loss from baseline^#^*	Temperature increase from baseline^#^*	Nasal inflammation indicators*	
				*Protein concentration*^#^	*Cell count*^#^
**0**	Weight increase or no change	≤20	<1	<500	<1X10^6^
**1**	1–3	21–40	1.1–1.5	500–1000	1 to 2 x10^6^
**2**	3–6	41–60	1.5–2.0	1001–1500	2.1 to 4 x10^6^
**3**	>6	>60	>2.0	>1500	>4x10^6^

*maximum values considered i.e. maximum weight loss, activity loss, protein concentration, etc

^#^ Weight loss and Activity loss are measured as % change from baseline, temperature measured in ͦC, protein content measured in μg/ml and cell count in cells/ml

**Table 3:** Severity of clinical signs in ferrets infected with influenza viruses.

Virus	Weight loss		Activity loss		Temperature		Nasal inflammation				Total Score	
							Protein		Cell			
	*Placebo*	*OST*	*Placebo*	*OST*	*Placebo*	*OST*	*Placebo*	*OST*	*Placebo*	*OST*	*Placebo*	*OST*
**Influenza A**												
**H1N1**	1	0	3	1	1	1	3	0	2	1	10	3
**H1N1(H275Y)**	1	1	2	3	3	2	2	0	1	1	9	7
**H1N1pdm09**	3	0	3	1	3	2	3	2	3	1	15	6
**H1N1pdm09(H275Y)**	3	3	3	3	3	3	3	3	3	3	15	15
**H3N2**	0	0	1	1	0	0	1	0	2	0	4	1
**H3N2(E119V)**	0	0	0	0	0	0	0	0	0	0	0	0
**Influenza B**												
**B**	0	0	1	1	2	0	1	1	0	1	3	3
**B(D197N)**	0	0	1	1	0	0	2	0	1	0	4	1
**B(H273Y)**	0	0	1	1	0	0	0	0	0	0	1	1

### Oseltamivir efficacy in ferrets infected with the H1N1 or H1N1(H275Y) virus

**Fig 1:**
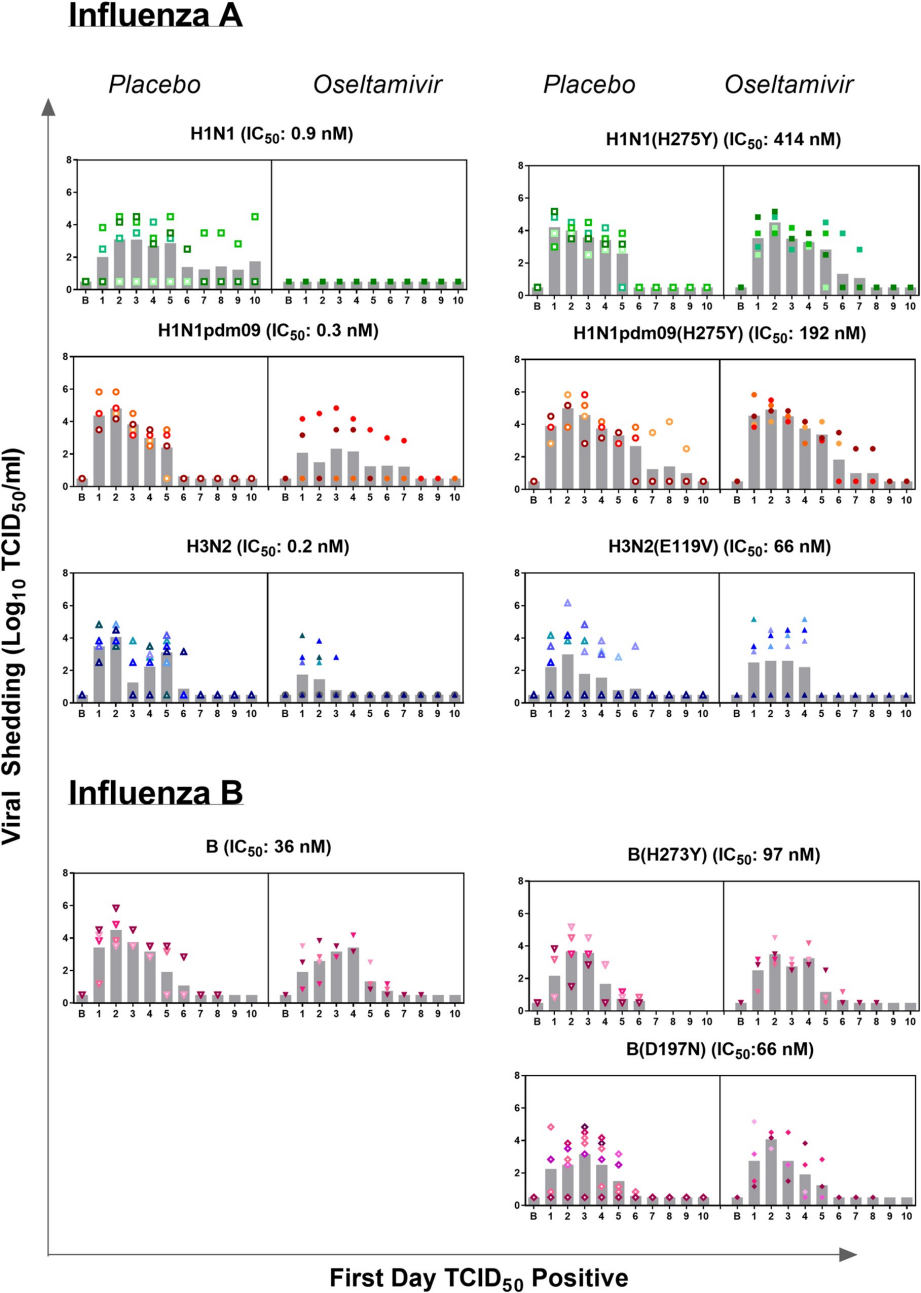
Viral shedding data from ferrets exposed to different viruses and dosed with either OST or Placebo. The data for all ferrets are shown after standardising to first day of TCID_50_ positive, as this day varied between different ferrets, and each individual ferret within a group is identified by a separate colour. The variability in first day of viral shedding is summarised in S2 Fig. The bar graphs represent the mean viral titre for all ferrets within the group on each day. The B stands for baseline.

OST was effective in preventing infectious virus shedding in all H1N1 exposed ferrets tested (0/4 infected), while 3/4 (75%) of the placebo dosed animals shed infectious virus for an average duration of 5.7 days (Fig 1, Table 1). The animals that did not shed infectious virus also remained seronegative for H1N1 (S1 Table).

Reductions in clinical signs were also apparent, with the placebo dosed ferrets that became infected with the H1N1 virus experiencing >50% loss in activity, whereas the OST dosed ferrets, who remained uninfected, had activity levels that fluctuated around pre-exposure baseline levels (S3 Fig). The difference in weight loss and nasal inflammation was also notable, with the difference in %AUC for weight, cell count and protein concentration being 2.5%, 67% and 74% respectively, and significant differences were observed in nasal inflammation indicators on Day 2 (S5 and S6 Figs). In contrast, OST was less effective against the H1N1 (H275Y) virus as all ferrets shed infectious virus, regardless of treatment group assigned. The clinical signs of the H1N1(H275Y) infected ferrets were somewhat improved due to OST (Table 3), and reduction in protein concentration in the nasal wash was significant on Day 4 (S6 Fig).

### Oseltamivir efficacy in ferrets infected with the H1N1pdm09 or H1N1pdm09 (H275Y) virus

Only 50% (2/4) of the OST dosed ferrets exposed to the H1N1pdm09 virus had detectable virus in their nasal washes, while all placebo dosed ferrets (4/4) became infected (Table 1). Of the two OST dosed ferrets that did not shed H1N1pdm09 virus, one developed a low HAI titre of 40, while the other remained seronegative (S1 Table). Amongst the ferrets that became infected, peak viral titres were similar between OST and placebo dosed animals (4.2±0.9 Log_10_ TCID_50_/ml vs. 4.8±0.7 Log_10_ TCID_50_/ml), however, on average, placebo dosed animals shed virus for a day longer (4.8±0.5 days), compared to OST dosed animals (3.5±0.7 days) (Table 1). The overall clinical score was substantially improved due to the OST dosing, with improvements seen across all parameters measured (Table 3), and significant differences observed in weight change, protein concentration and activity levels on Day 4 (S4–S6 Figs), with differences in %AUC being 4.9%, 56% and 53%, respectively. In ferrets exposed to the H1N1pdm09 (H275Y) virus, OST dosing had little benefit compared to placebo, as all virological parameters and clinical score remained similar between OST dosed and placebo dosed animals.

### Oseltamivir efficacy in ferrets infected with the H3N2 or H3N2 (E119V) virus

OST was effective at preventing 4/8 (50%) ferrets from shedding infectious virus following exposure to the H3N2 virus, whereas all exposed placebo ferrets (7/7) shed detectable virus (Table 1). Of note, the four OST dosed animals that did not shed infectious virus, nevertheless seroconverted with HI titres ranging between 80–160 (S1 Table). The OST dosed ferrets that became infected with the H3N2 virus had a significantly lower peak viral titre (4.4±0.4 Log_10_ TCID_50_/ml vs. 3.0±0.8 Log_10_ TCID_50_/ml) and duration of viral shedding (5.0±0.6 vs 2.0±0.8 days) than placebo dosed ferrets (Fig 1, Table 1). The magnitude of clinical signs in ferrets infected with the H3N2 viruses was lower than those ferrets infected with either the H1N1 or H1N1pdm09 virus (Table 3). As such the efficacy of OST on weight, activity or temperature change in ferrets infected with the H3N2 virus was minimal (Table 3). However, OST dosing did reduce nasal inflammation in H3N2 infected ferrets, with significant differences in cell counts observed on most days (S5 Fig), and significant difference in protein concentrations observed on Day 7 and 8 (S6 Fig). The %AUC for cell counts and protein concentration were reduced by 69.2% and 52.4% respectively.

Similar to previous results, OST efficacy in preventing or reducing viral shedding was lost against the H3N2 virus with the E119V substitution, and clinical signs overall were minimal, making comparisons between groups difficult.

### Oseltamivir efficacy in ferrets infected with the influenza B, B (D197N) or B (H24Y) virus

Although OST was ineffective at preventing infection in ferrets exposed to the B virus, peak viral titre and AUC tended to be lower in the OST dosed ferrets compared to placebo dosed ferrets, although it wasn’t significantly different (Table 1). Due to the short timeframe of the experiment (10 days), influenza B infected animals did not seroconvert (but may have if tested later). Clinical signs were very also mild in ferrets infected with the B viruses and no notable OST effect was observed on activity, weight, temperature or nasal inflammation (Table 3).

Similar to wild-type B virus, OST dosing did not reduce infection rates in ferrets exposed to either B(D197N) or B(H273Y) viruses. Comparison of viral titres between placebo and OST dosed ferrets across each day also revealed no significant differences, except for a single day when OST dosed ferrets infected with the B (D197N) virus shed viruses at a significantly higher titre (4.1 ± 0.4 Log_10_ TCID_50_/ml) than their placebo counterparts (2.5 ± 1.3 Log_10_ TCID_50_/ml). Clinical signs were also mild in B(D197N) or B(H273Y) infected ferrets. No difference in activity, weight, and cell count were observed between ferrets infected with the B (H273Y) virus dosed with either OST or placebo (S3–S5 Figs). Ferrets infected with the B (D197N) virus did not experience any weight loss, and activity was similar between OST and placebo ferrets (S3 and S4 Figs). However, cell counts and protein concentrations were generally higher in the nasal washes of ferrets infected with the B (D197N) virus and dosed with placebo, with a significant difference in protein concentrations observed on Day 7 (S6 Fig, Table 3).

Genetic analysis of nasal washes revealed some changes in the HA and NA genes of the all the test viruses, summarised in detail in S2 Table (S2 Table). However, none of the genetic changes in the NA were in the active site or have been previously associated with reduced susceptibility to OST.

### Correlating the *in vitro* IC_50_ values with the *in vivo* effects of oseltamivir

**Fig 2:**
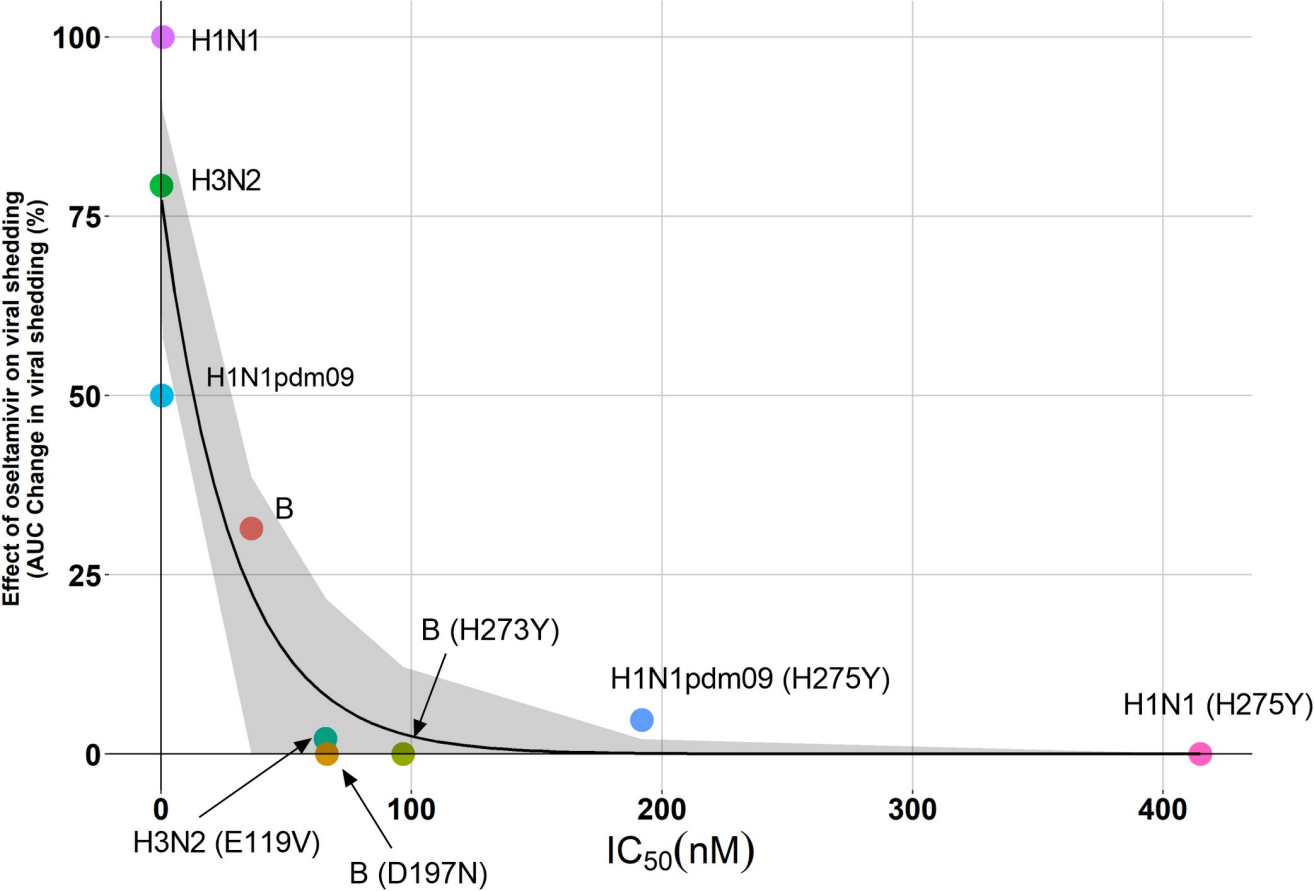
A scatter plot showing the relationship between %ΔAUC (difference between placebo and OST dosed animals) of viral shedding of ferrets and the OST IC_50_. This graph shows the significant correlation between in vitro OST IC_50_ and *in vivo* effect on viral shedding. The regression model reveals the following relationship where y = e^4.35–0.03x^. The shaded region of the graph is the 95% confidence interval of the line of best fit, calculated by bootstrapping over 2500 iterations.

The IC_50_ values calculated from a neuraminidase inhibition assay, measures OST’s capacity to inhibit the enzymatic activity of the influenza NA protein. It was our aim to correlate values from this *in vitro* assay with a more biologically relevant metric. The relative change in Area Under the Curve in viral shedding curves (%ΔAUC), comparing placebo dosed and OST dosed animals, was chosen as such a metric, as it summarises key virological parameters including peak viral titres, infection rate, and duration of viral shedding in the one measurement. Fig 2 reveals a non-linear relationship between IC_50_ and %ΔAUC of viral shedding, with a Spearman correlation coefficient of -0.81 (p = 0.017). This non-linear regression model could then be used to estimate that an IC_50_ of 14.6 nM, corresponded to a 50% reduction in OST efficacy and an IC_50_ of 68.2 nM corresponded to a 90% loss in oseltamivir efficacy against viral shedding. Effectiveness of OST in recovering activity trended with IC_50_ values although Spearman’s rho was only -0.48 and not significant (p = 0.194). A significant correlation (rho = -0.7, p = 0.043) between weight changes and IC_50_ values was observed. However, a %ΔAUC in weight change was observed even when placebo animals did not lose weight, due to the weight gain of their OST counterparts, and as such this correlation does not truly reflect OST’s effect in recovering weight loss. Temperature, cell count and protein concentration were not well correlated with IC_50_ values (spearman rhos were -0.1, -0.41 and -0.18 respectively).

### OST efficacy in macaques

**Fig 3:**
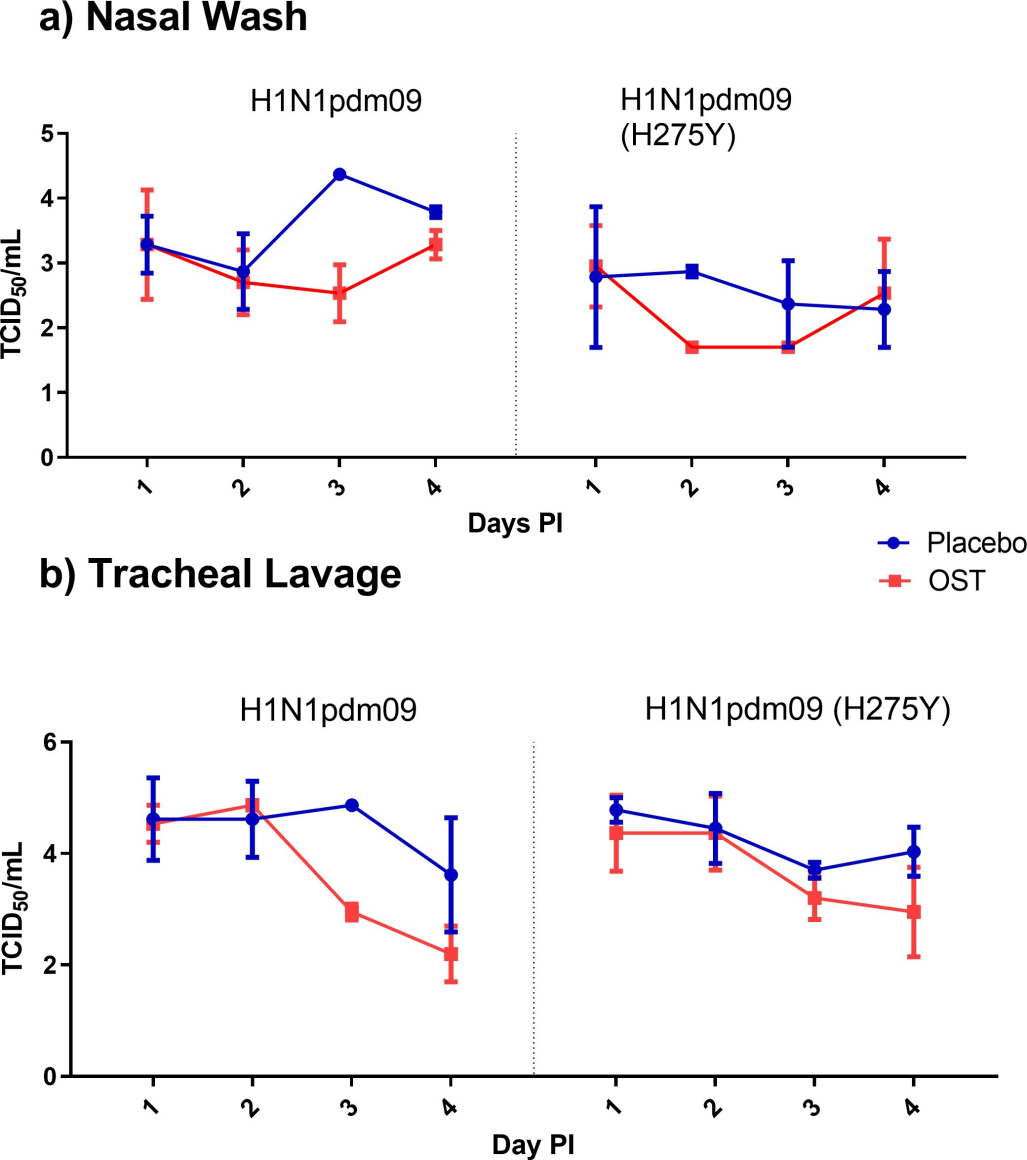
Influenza virus titres from nasal washes and tracheal lavages from macaques infected with either H1N1pdm09 or H1N1pdm09 (H275Y) virus and treated with either OST or placebo. Three macaques were allocated per group and artificially infected with 106 TICD_50_/ml of viral inoculum. No significant differences were observed in viral titre between animals treated with OST compared to those treated with placebo. Panel a) shows viral titre data from nasal washes of animals and b) shows viral tire data from tracheal lavages.

To evaluate if the findings in ferrets would also be observed in an animal model that has more similar lung physiology and drug metabolism to that of humans, a macaque model of infection was used [58, 59]. Animals were infected with either the H1N1pdm09 virus or the H1N1pdm09 (H275Y) virus and OST dosing was compared to placebo. In this study, the macaques did not develop any clinical signs, and there were no significant differences in body weight or temperature between OST- and placebo-treated animals. Viral titres in nasal wash and tracheal lavages were low (ranging between 10^3^−10^5^ TCID_50_/ml) and not significantly different between placebo and OST dosed macaques (Fig 3). It should be noted however that, in animals infected with the H1N1pdm09 virus, viral titres were 1.9 logs lower in the tracheal lavages of OST dosed macaques compared to placebo animals, on Day 3 post-infection. A similar phenomenon was not observed in animals infected with the H1N1pdm09(H275Y) virus.

Sequence analysis of viruses recovered from the tracheal lavage did not indicate reversion of the H275Y substitution in NA and only minor changes in the rest of the NA and HA genes (S2 Table).

## Discussion

In this study the efficacy of OST was evaluated in the ferret model against nine different influenza viruses with OST IC_50_ values ranging from 0.2–414 nM. OST efficacy was evaluated based on assessment of virological parameters and clinical signs of infection. We were able to show that OST was effective in reducing viral shedding in the upper respiratory tract of ferrets infected with influenza A wild-type viruses. However, this drug effect was reduced in animals infected with wild-type influenza B virus and influenza A or B viruses with increased IC_50_ levels.

This study also aimed to investigate the efficacy of OST in a macaque model challenged with an A(H1N1)pdm09 wild type or a H275Y bearing virus. However, a lack of clinical symptoms and lower virus titres in respiratory samples made it difficult to show any significant reductions with OST. Previous studies have shown that the clinical signs of A(H1N1)pdm09 virus infection in macaques can be dependent on virus strains [60–62]. It is therefore important to further develop the macaque model of OST treatment, possibly using different viral strains, to reach a more conclusive result regarding efficacy. While the conclusions from this part of the study are limited, this data adds to the small pool of literature available on NAI efficacy in the macaque influenza challenge model [63–67].

In this study, we were able to establish a significant correlation between *in vitro* generated OST IC_50_ values and the efficacy of OST in reducing viral shedding *in vivo* in ferrets. We found that IC_50_ can be a strong predictor of OST efficacy, regardless of virus background. For example, the viral replication of the H3N2(E119V) and the B(D197N) viruses, which had similar IC_50_ values (ranging between 65-66nM), were similarly impacted by OST treatment.

OST was less effective in reducing the severity of clinical signs in ferrets infected with wild-type influenza B virus, compared to those infected with wild-type influenza A viruses, although this was in part due to the clinical signs from the influenza B infection being milder than those with the influenza A viruses. A number of clinical trials have also shown OST treatment to be less effective in reducing symptom duration and severity in patients infected with influenza B viruses compared to those infected with influenza A viruses [34–39, 68]. Our finding, combined with these previous results, provides increased evidence that OST has reduced efficacy against wild-type influenza B viruses. This has implications for how influenza B viruses with reduced drug susceptibility are defined and understood. For example, the influenza B (H273Y) virus in this study has a 3-fold increase in IC_50_ compared to its corresponding wild-type, and under the current WHO guidelines would be classified as having ‘normal inhibition’, even though our study shows that OST efficacy in reducing viral shedding of this virus was lower than that of wild-type influenza B. These findings suggest that the WHO criteria for defining influenza B viruses with ‘reduced’ or ‘highly reduced inhibition’ may need to be revisited. However more human clinical data needs to be collected regarding OST efficacy against influenza B viruses with reduced *in vitro* susceptibility before a decision on any new criteria should be made.

A limitation of the model is that different viruses confer different pathogenicity in ferrets, which makes evaluating OST efficacy based on clinical signs more difficult. The clinical score for infection was lower for H3N2 and B infections than H1N1 or H1N1pdm09 infections, making it more difficult to evaluate OST efficacy against these viruses. Viruses with reduced NAI susceptibility can also exhibit reduced pathogenicity which can make evaluating the clinical effect of antiviral treatment against these viruses challenging. Whilst this study demonstrated that the mild clinical signs in untreated ferrets resulted in insufficient capacity to detect an effect of OST, the effect of OST treatment was able to elicit a strong correlation between viral shedding and OST IC_50_. Host factors, such as ferret immunity (they are naïve to influenza), are an additional caveat to the analysis in this study, further exacerbated by small ferret numbers (ranging between 4–8 animals/group), which limits the power of statistical analysis. While improving the power of this study would be desirable, the use of larger animal cohorts is prohibitively expensive, and insights derived from small group sizes of n = 4 animal numbers have previously provided valuable insights [40, 43, 45, 69–75]. Finally, another limitation of this study is that the results observed in ferrets and macaques may not directly align with those seen in humans, as they not only have physiological differences but in most cases (apart from small infants) humans have some level of prior immunity to influenza [40, 41, 43, 45, 69–76]. However even with these limitations, data from animal models remains very useful, and have been previously used to gain valuable insights into influenza virus biology and antiviral efficacy [40, 41, 43, 45, 69–76].

The ferret treatment model used in this study involved the initiation of OST prior to influenza infection, whereas antiviral treatment in humans is typically initiated 24 to 48 hours after infection or onset of symptoms. In addition, in the ferret model dosing was maintained for 10 days as opposed to 5 days in the typical human situation [77]. We have previously conducted studies to evaluate the effect of OST when it was initiated 12 or 24-hours post-exposure but little or no virological effect was observed making that protocol unsuitable for testing of OST [47]. To optimise the virological effect of oseltamivir in the ferret, we modified the model such that ferrets were infected by exposure to an infected donor, instead of direct intranasal inoculation. This method has the drawback that the exact time of infection for each ferret is unknown. Therefore, dosing was standardized to start 2-hours prior to exposure for all animals, and as some animals became influenza virus positive late in the experiment (Day 6 or 7, S1 Fig), dosing was continued throughout the full length of the experiment (10 days). Therefore, whilst the dosing regimen used in the ferrets differs from the standard human treatment regimen, it was necessary to change these aspects to achieve a ferret model where an OST effect was observed allowing subsequent evaluation of the impact of OST on viruses with reduced susceptibility.

In summary, we investigated the relationship between *in vitro* IC_50_ and *in vivo* efficacy of OST in two complementary animal models. While data from the macaque model was not conclusive, we were able to show in ferrets that OST was less effective in reducing viral shedding against viruses with higher IC_50_ values than wild-type viruses. It should be noted that while IC_50_ was shown to be an important predictive measure, viral pathogenicity, and immunological factors can also play a role in OST efficacy. This study is the first to quantitatively illustrate how OST efficacy correlates to the increased IC_50_ of human influenza viruses, and highlights the need to re-evaluate the current guidelines for defining ‘normal’, ‘reduced’ or ‘highly reduced’ inhibition for influenza B viruses, although further human clinical studies and analysis are needed to establish more relevant levels.

## Supporting information

S1 Fig Experimental model used to assess efficacy of OST against viruses in ferrets. (DOCX)

S2 Fig Summary of variability in first day of TCID_50_ positivity of individual ferrets within a group. (DOCX)

S3 Fig Change in % activity of ferrets exposed to different viruses and dosed with either OST or Placebo. (DOCX)

S4 Fig Summary of change in % weight of ferrets exposed to different viruses and dosed with either OST or Placebo. (DOCX)

S5 Fig Summary of change in cell count in nasal wash of ferrets exposed to different viruses and dosed with either OST or Placebo. (DOCX)

S6 Fig Summary of change in protein concentration in nasal wash of ferrets exposed to different viruses and dosed with either OST or Placebo. (DOCX)

S7 Fig Summary of change in temperature of ferrets exposed to different viruses and dosed with either OST or Placebo. (DOCX)

S1 Table Serological data of ferrets exposed to influenza A viruses. (DOCX)

S2 Table Summary of influenza variants arising in ferrets and macaques following replication. (XLSX)
