# Supplementary material for: Utilising animal models to evaluate oseltamivir efficacy against influenza A and B viruses with reduced in vitro susceptibility
Source: PLoS Pathog. 2020 Jun 18;16(6):e1008592. doi: 10.1371/journal.ppat.1008592 (PMC7326275; doi:10.1371/journal.ppat.1008592)
Supplement: S1 Fig — (DOCX) [file ppat.1008592.s001.docx]

**Figure S1: Model Setup**


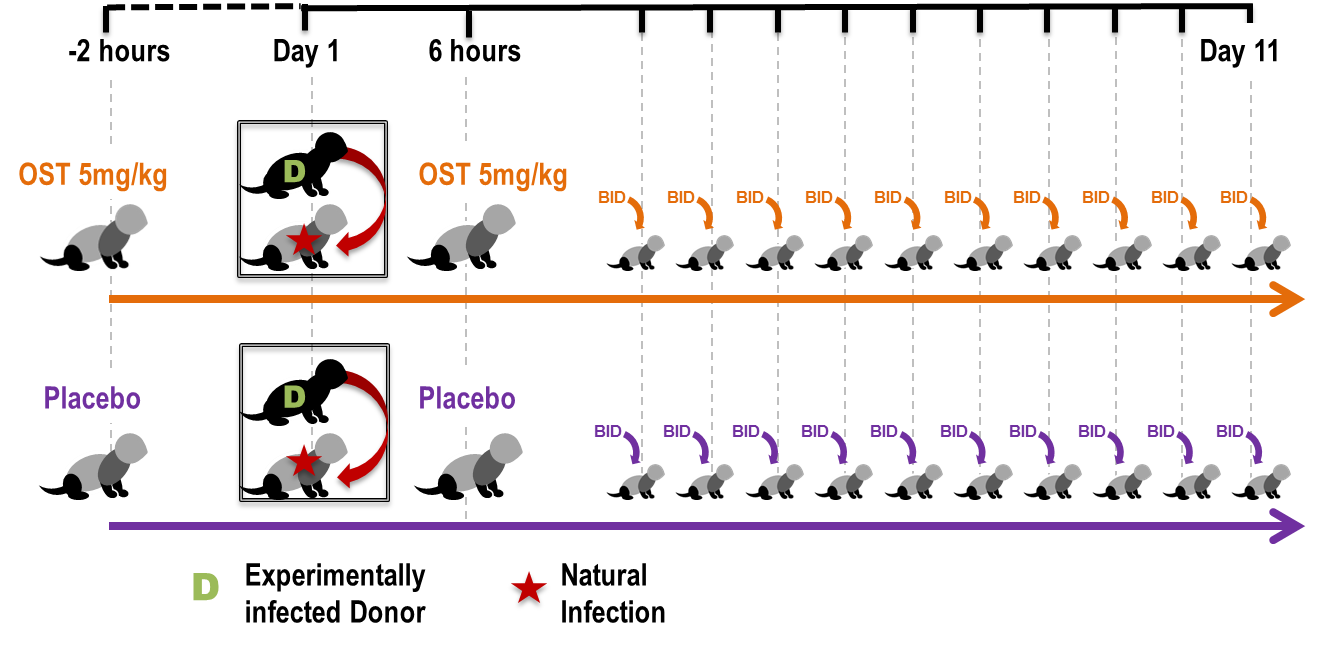
**Figure S1: Experimental model used to assess efficacy of OST against viruses in ferrets.** Test ferrets (grey) were given either 5mg/kg of OST or Placebo, 2-hours prior to introduction to an experimentally infected donor (black). The donor was infected a day prior with an experimental inoculum of 10^6^ TCID_50_/ml of viruses. The test ferrets were then dosed 6-hours post co-housing with either OST or placebo again. The next 10 days, the test ferrets were dosed twice daily (BID: *bis in die*) with either OST or placebo. Length of co-housing with donor ferrets varied for each virus; for donors infected with H1N1 or H1N1 (H275Y) it was 2 days, for donors infected with H3N2 or H3N2 (E119V) it was 6 days and for donors infected with B, B (H273Y) or B (D197N) it was 11 days. Throughout the length of the experiments test ferrets were monitored for weight, temperature and activity. Daily nasal wash also collected from the test ferrets.
