# Supplementary material for: Utilising animal models to evaluate oseltamivir efficacy against influenza A and B viruses with reduced in vitro susceptibility
Source: PLoS Pathog. 2020 Jun 18;16(6):e1008592. doi: 10.1371/journal.ppat.1008592 (PMC7326275; doi:10.1371/journal.ppat.1008592)
Supplement: S2 Fig — (DOCX) [file ppat.1008592.s002.docx]

**Figure S2: Day to first positive infection**


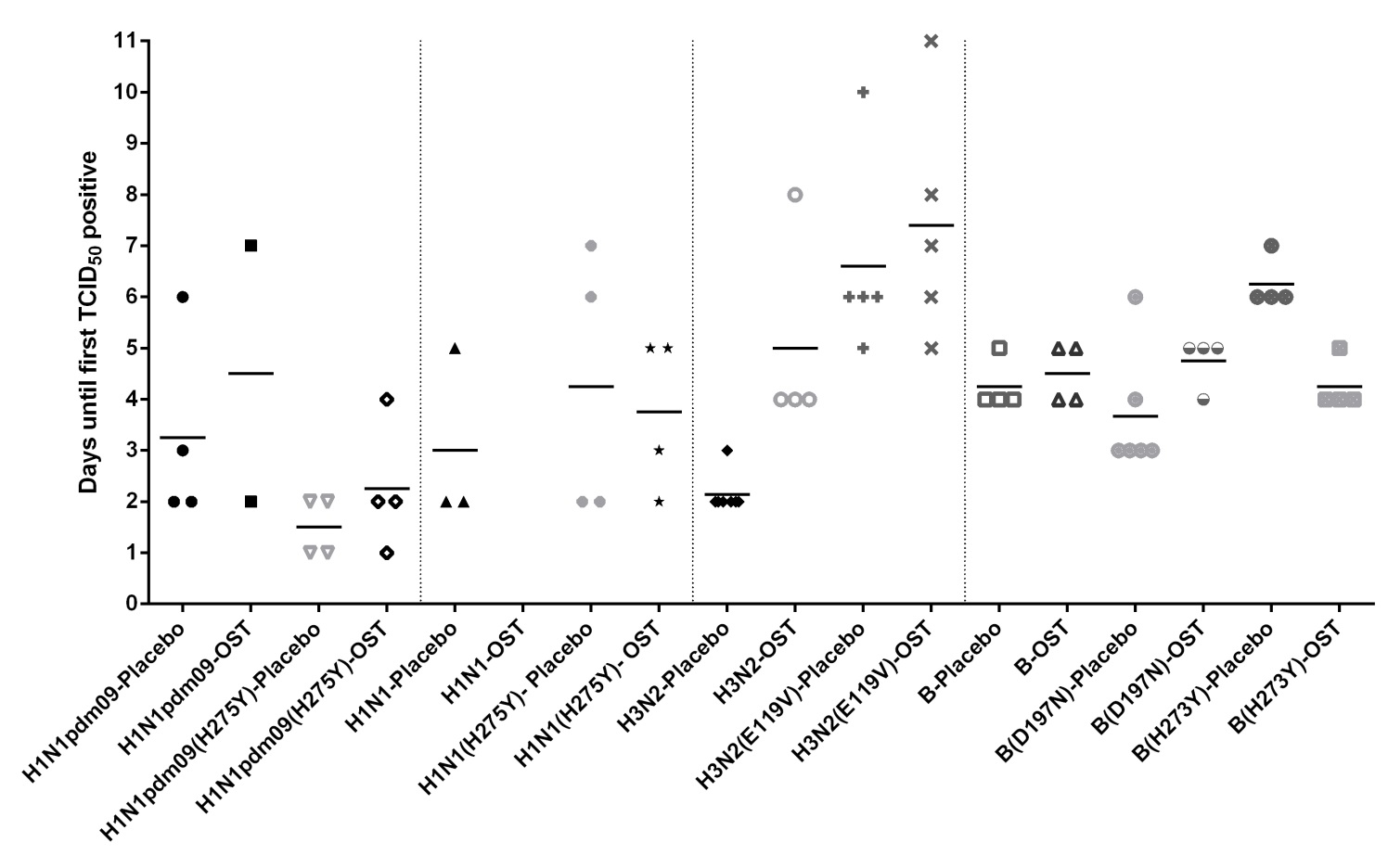


**Figure S2: Summary of variability in first day of TCID_50_ positivity of individual ferrets within a group.** The mean for the ferrets within a group is denoted by the horizontal line. A large variability was seen even within groups, with values ranging from 1-5 days. The variability in time of infection was smaller for ferrets infected with influenza B, B (D197N) or B(H273Y). Ferrets in the H3N2 (E119V) group showed the longest average time to first day of viral shedding.
