# Supplementary material for: Utilising animal models to evaluate oseltamivir efficacy against influenza A and B viruses with reduced in vitro susceptibility
Source: PLoS Pathog. 2020 Jun 18;16(6):e1008592. doi: 10.1371/journal.ppat.1008592 (PMC7326275; doi:10.1371/journal.ppat.1008592)
Supplement: S3 Fig — (DOCX) [file ppat.1008592.s003.docx]

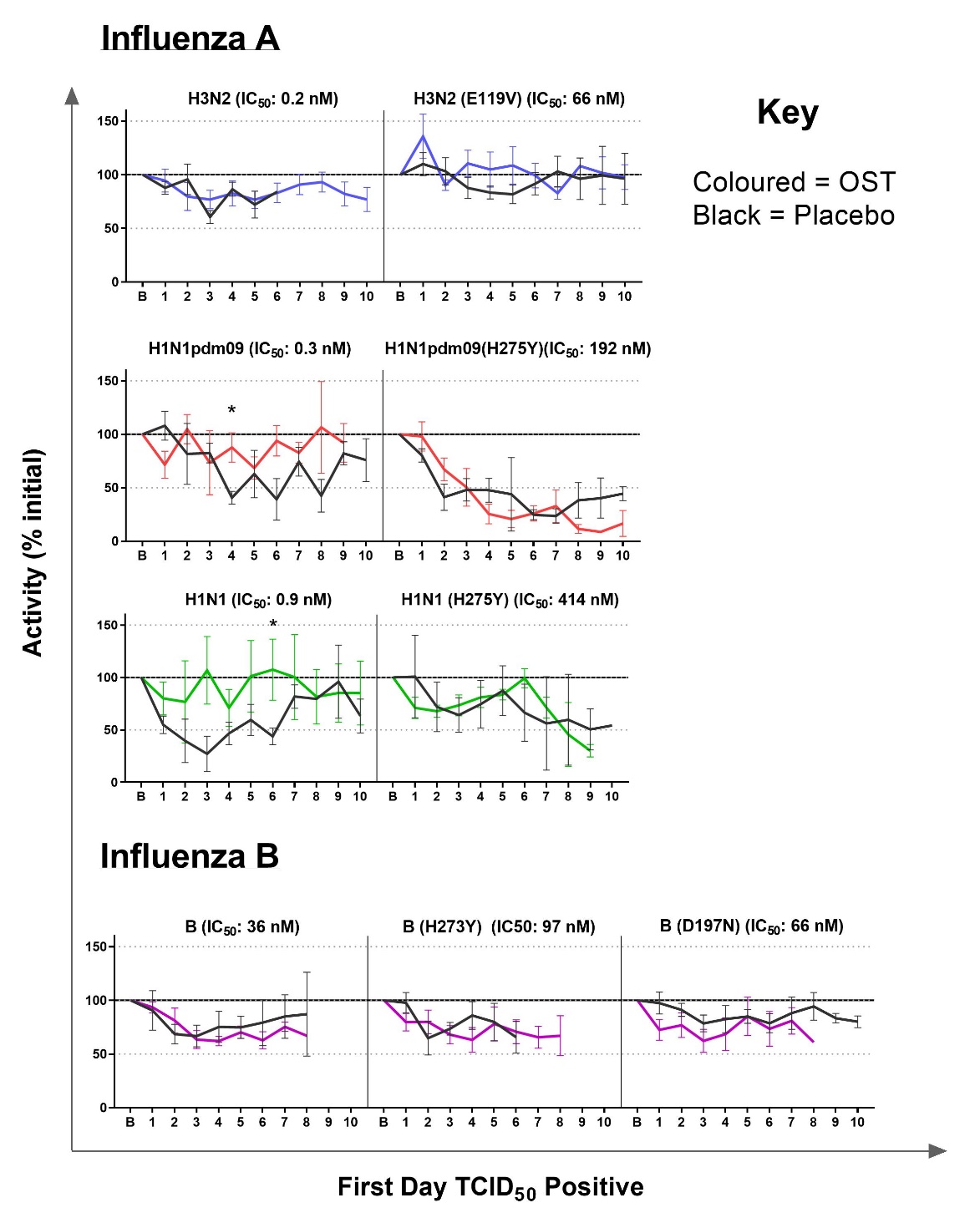


**Figure S3: Change in % activity of ferrets exposed to different viruses and dosed with either OST or Placebo.** Three days prior to start of experiment, ferret activity was measured using video monitoring to establish baseline activity levels (B), which was set at 100%, and activity level was measured each day during experiment. Video-monitoring data was analysed using EthoVision software as previously described and the activity for each ferret was calculated as a percentage of their baseline. The line plot in this figure shows mean % Activity ± SEM for all ferrets in a group. If significant differences are observed between OST dosed and placebo dosed animals on an individual day, a ‘*’ is used to denote that.
