# Supplementary material for: Utilising animal models to evaluate oseltamivir efficacy against influenza A and B viruses with reduced in vitro susceptibility
Source: PLoS Pathog. 2020 Jun 18;16(6):e1008592. doi: 10.1371/journal.ppat.1008592 (PMC7326275; doi:10.1371/journal.ppat.1008592)
Supplement: S6 Fig — (DOCX) [file ppat.1008592.s006.docx]

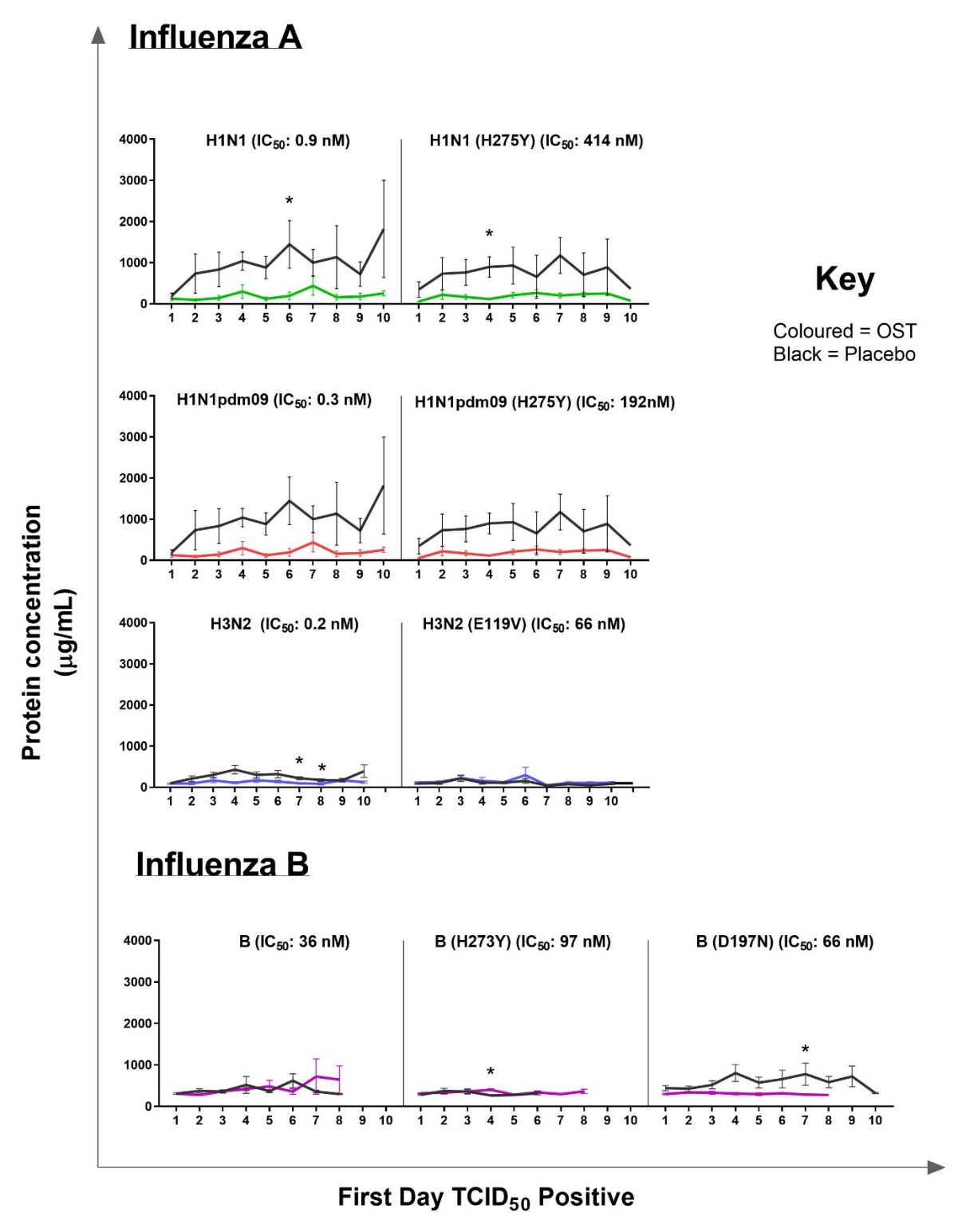


**Figure S6: Summary of change in protein concentration in nasal wash of ferrets exposed to different viruses and dosed with either OST or Placebo.** Nasal wash was collected each day and protein concentration was measured using the Bradford assay. The bar graphs in this figure show mean protein concentration ± SEM for all ferrets in a group. If significant differences are observed between OST dosed and placebo dosed animals on an individual day, a ‘*’ is used to denote that.
