# Supplementary material for: Utilising animal models to evaluate oseltamivir efficacy against influenza A and B viruses with reduced in vitro susceptibility
Source: PLoS Pathog. 2020 Jun 18;16(6):e1008592. doi: 10.1371/journal.ppat.1008592 (PMC7326275; doi:10.1371/journal.ppat.1008592)
Supplement: S7 Fig — (DOCX) [file ppat.1008592.s007.docx]

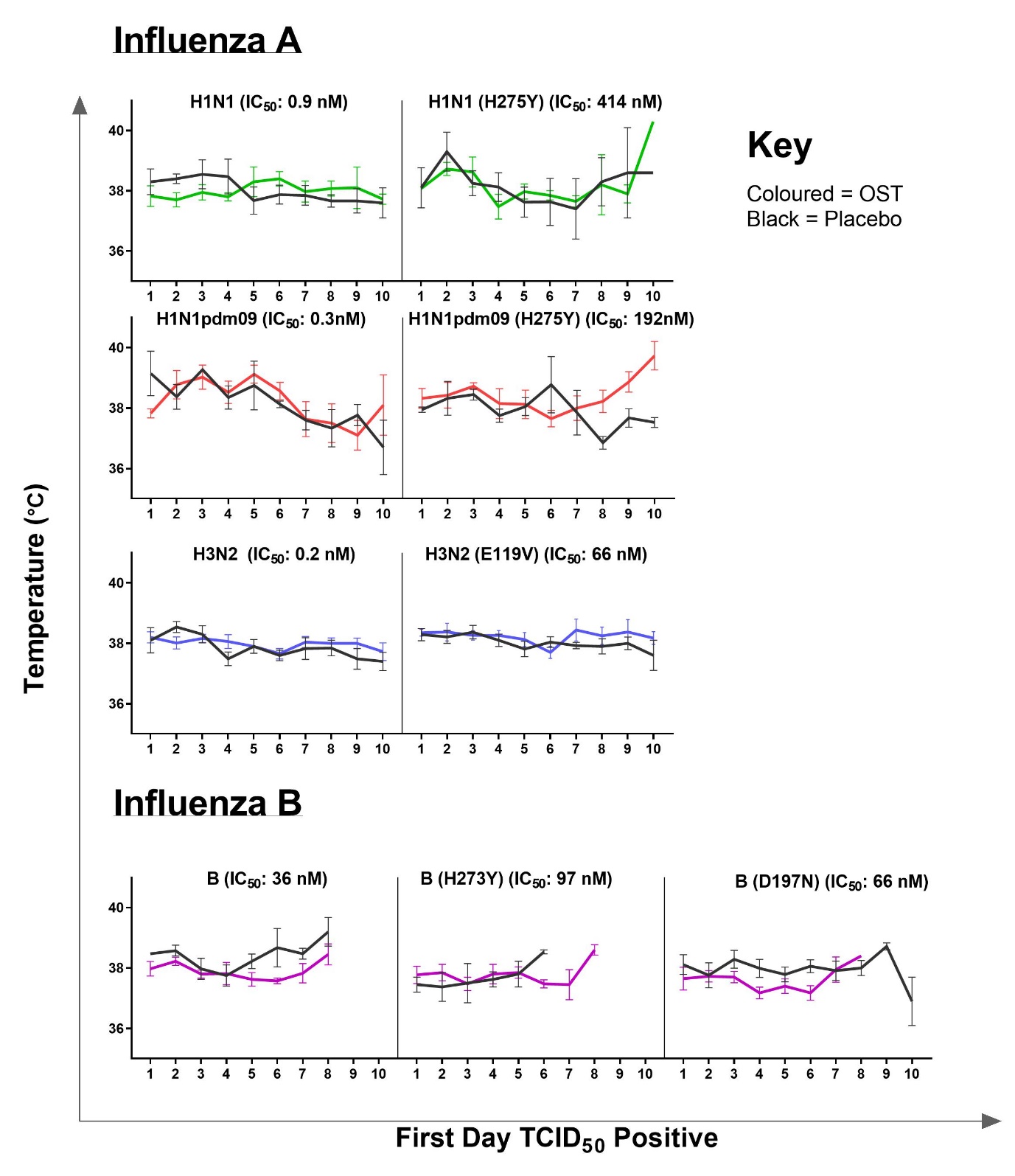


**Figure S7: Summary of change in temperature of ferrets exposed to different viruses and dosed with either OST or Placebo.** The bar graphs in this figure show mean temperature ± SEM for all ferrets in a group. If significant differences are observed between OST dosed and placebo dosed animals on an individual day, a ‘*’ is used to denote that.
