# Supplementary material for: Utilising animal models to evaluate oseltamivir efficacy against influenza A and B viruses with reduced in vitro susceptibility
Source: PLoS Pathog. 2020 Jun 18;16(6):e1008592. doi: 10.1371/journal.ppat.1008592 (PMC7326275; doi:10.1371/journal.ppat.1008592)
Supplement: S1 Table — (DOCX) [file ppat.1008592.s008.docx]

|  | Placebo | | | | | | | | OST | | | | | | | |
| --- | --- | --- | --- | --- | --- | --- | --- | --- | --- | --- | --- | --- | --- | --- | --- | --- |
|  |  |  |  |  |  |  |  |  |  |  |  |  |  |  |  |  |
| H1N1 Ferret #^a^ | 46296 | 46324 | 46312 | 82727 |  |  |  |  | 46331 | 46327 | 46315 | 82627 |  |  |  |  |
| HI Titre | 40 | 40 | - | 60 |  |  |  |  | - | - | - | - |  |  |  |  |
| Viral Shedding | + | + | - | + |  |  |  |  | - | - | - | - |  |  |  |  |
|  |  |  |  |  |  |  |  |  |  |  |  |  |  |  |  |  |
| H1N1 (H275Y) Ferret # | 46299 | 46300 | 82729 | 82694 |  |  |  |  | 46829 | 46333 | 46301 | 46304 |  |  |  |  |
| HI Titre | - | - | 40 | - |  |  |  |  | 40 | 60 | 20 | - |  |  |  |  |
| Viral Shedding | + | + | + | + |  |  |  |  | + | + | + | + |  |  |  |  |
|  |  |  |  |  |  |  |  |  |  |  |  |  |  |  |  |  |
| H1N1pdm09 Ferret # | 82723 | 82724 | 46313 | 46329 |  |  |  |  | 82730 | 82691 | 46307 | 82689 |  |  |  |  |
| HI Titre | 20 | 20 | 20 | 20 |  |  |  |  | - | 40 | - | 40 |  |  |  |  |
| Viral Shedding | + | + | + | + |  |  |  |  | + | + | - | - |  |  |  |  |
|  |  |  |  |  |  |  |  |  |  |  |  |  |  |  |  |  |
| H1N1pdm09 (H275Y) Ferret # | 82683 | 82728 | 46302 | 46310 |  |  |  |  | 46311 | 46330 | 46303 | 46244 |  |  |  |  |
| HI Titre | 40 | 20 | 20 | 40 |  |  |  |  | 20 | 20 | 40 | - |  |  |  |  |
| Viral Shedding | + | + | + | + |  |  |  |  | + | + | + | + |  |  |  |  |
|  |  |  |  |  |  |  |  |  |  |  |  |  |  |  |  |  |
| H3N2 Ferret # | 46891 | 48487 | 48348 | 8719 | 8801 | 10143 | 9690 |  | 48353 | 48352 | 48350 | 48351 | 15517 | 10811 | 9070 | 9272 |
| HI Titre | - | 160 | 40 | - | 20 | - | 20 |  | 160 | 160 | 160 | 160 | 160 | 40 | 80 | 80 |
| Viral Shedding | + | + | + | + | + | + | + |  | + | + | - | - | - | + | + | - |
|  |  |  |  |  |  |  |  |  |  |  |  |  |  |  |  |  |
| H3N2 (E119V) Ferret # | 46894 | 46918 | 48355 | 48354 | 13183 | 10710 | 13400 | 14792 | 48357 | 46889 | 46888 | 48356 | 95377 | 12829 | 9050 | 15150 |
| HI Titre | 20 | 20 | 20 | - | 40 | 20 | - | - | 160 | 40 | 80 | 80 | 80 | 80 | 80 | 160 |
| Viral Shedding | - | + | - | + | - | + | + | + | + | + | + | - | - | + | - | + |

**Table S1:** **Serological data of ferrets exposed to influenza A viruses**^b^

^a^ Ferret Chip number used to delineate each individual ferret

^b^ Influenza B viruses showed no seroconversion as 10 days was not enough to develop an antibody response for influenza B
